# Developing a practice framework for patient navigation in cancer care: a Global Initiative to Advance Cancer Navigation for Better Outcomes (GINO) project

**DOI:** 10.1016/j.eclinm.2026.103808

**Published:** 2026-02-23

**Authors:** Imogen Ramsey, Fiona Crawford-Williams, Carla Thamm, Dawn Aubel, Jacqueline L. Bender, Alexandre Chan, Melissa Chin, Margaret I. Fitch, Michael Jefford, Ebele Mbanugo, Enrique Soto-Perez-de-Celis, Carolyn Taylor, Raymond J. Chan, MASCC GINO Patient Navigation Working Group, Oluwaseyifunmi Andi Agbejule, Muna Alkhaifi, Cristiane Bergerot, Darcy Burbage, Andreai Capela, Yin Ting Cheung, Niharika Dixit, Carolyn Ee, Kristen Haase, Nicolas H. Hart, Darren Haywood, Ria Joseph, Debbie Kirk, Stefano Magno, Aalaa Mahmoud, Duska Petranovic, Dagmara Poprawski, Emad Shash, Sandra Sonego

**Affiliations:** a Caring Futures Institute, Flinders University, Adelaide, South Australia, Australia; b Novartis, Basking Ridge, NJ, USA; c Cancer Rehabilitation and Survivorship, Department of Supportive Care, Princess Margaret Cancer Centre, Toronto, Ontario, Canada; d Dalla Lana School of Public Health, University of Toronto, Toronto, Ontario, Canada; e Department of Clinical Pharmacy Practice, University of California, Irvine, Irvine, CA, USA; f Multinational Association for Supportive Care in Cancer, Toronto, Ontario, Canada; g Bloomberg Faculty of Nursing, University of Toronto, Toronto, Ontario, Canada; h Australian Cancer Survivorship Centre, Peter MacCallum Cancer Centre, Melbourne, Victoria, Australia; i Department of Health Services Research, Peter MacCallum Cancer Centre, Melbourne, Victoria, Australia; j Sir Peter MacCallum Department of Oncology, University of Melbourne, Parkville, Victoria, Australia; k Run for a Cure Africa, Lagos, Lagos State, Nigeria; l University of Colorado Cancer Centre, University of Colorado Anschutz Medical Campus, Aurora, CO, USA; m Global Focus on Cancer, South Salem, NY, USA

**Keywords:** Cancer navigation, Oncology navigation, Patient navigation, Practice framework, Delphi study

## Abstract

Patient navigation comprises person-centred activities focused on addressing barriers and facilitating timely access to health care. Despite demonstrated effectiveness, the scope of patient navigation remains unclear. To clarify the scope of patient navigation and support global implementation, the Global Initiative to Advance Cancer Navigation for Better Outcomes (GINO) aimed to develop a practice framework for patient navigation in cancer care. Phase 1 involved reviewing patient navigation literature and identifying key areas of practice. Phase 2 involved a modified Delphi process with international experts in navigation (July to December 2024) to establish consensus on practices to include in the framework. Patient navigation experts across regions and disciplines were invited. Two rounds of online surveys were conducted where participants rated the importance of each practice on a scale from 1 (“Not important”) to 5 (“Critically important”). Practices rated ≥4 by ≥ 75% of participants in Round 2 met consensus criteria. The remaining items were discussed in a consensus meeting. Eighty-one experts from 29 countries (n = 45, 56% high-income, n = 36, 44% low-middle-income) participated in Round 1. Of these, 60 also participated in Round 2, including healthcare practitioners (n = 30, 50%), navigators (n = 16, 27%), researchers (n = 24, 40%), and advocates (n = 10, 17%). After Round 2, 27/35 (77%) practices reached consensus for inclusion. After the consensus meeting, two items were reworded, and 32 items were included in the final framework. We reached consensus among international experts on the contents of the GINO practice framework for patient navigation in cancer care. By describing the scope of patient navigation, the framework can support the development and implementation of patient navigation programs globally.

## Introduction

The concept of patient navigation in health care originated in the United States (US) in 1990 as a community-based intervention to help socially and economically marginalised individuals access timely cancer screening, diagnosis, and treatment.^1^ By providing individualised support, patient navigation aimed to help address individual, organisational, and systemic barriers to care.^1^ Over the past three decades, extensive research has demonstrated the effectiveness of patient navigation in reducing disparities in cancer care.^2^ An umbrella review of 61 systematic reviews found emerging to strong evidence that patient navigation is associated with improved cancer screening rates, reduced time to diagnosis, decreased hospital readmissions, increased adherence to follow-up appointments, improved decision-making and knowledge among cancer survivors, higher patient satisfaction with care, and improved quality of life.^3^ Although patient navigation is applicable across health settings and disease types, the concept originated in oncology and is particularly relevant in cancer care due to the complexity of oncology pathways, the multiple providers and care settings involved and the psychosocial impacts.^1^

Patient navigation is broadly defined as “an individualised intervention that aims to address barriers and facilitate timely access to healthcare services, diagnosis, treatments and care.”^3^ The scope, activities, and qualifications of individuals who provide patient navigation vary significantly, creating challenges for research, practice, and policy due to the lack of consistent role definitions.^4^^,^^5^ For example, navigation services may be provided by trained non-clinical navigators or individuals with professional clinical qualifications, such as nurses and social workers.^1^ Across different health systems, individuals fulfilling a clinical navigator role have also been identified by a variety of titles, including clinical or cancer care coordinators, case managers, cancer support nurses, and follow-up nurses.^5^

Following recommendations by Freeman and Rodriguez (2011) to define the scope of patient navigation,^1^ several initiatives have sought to distinguish navigators’ roles and responsibilities and standardise delivery of patient navigation. In 2012, Canadian researchers developed the patient navigation framework,^6^ conceptualising navigation as a bi-dimensional process aimed at facilitating continuity of care and promoting empowerment.^6^ In 2013, the Oncology Nursing Society published core competencies and a professional practice framework for oncology nurse navigators.^7^ These were updated in 2018 and again in 2024 to reflect the evolution of the role in the changing oncology environment.^8^ In parallel, the George Washington Cancer Institute developed a patient navigation framework delineating roles and responsibilities across patient navigators (clinically and non-clinically licenced) and community health workers,^9^^,^^10^ as well as competencies for oncology patient navigators.^10^^,^^11^ In a significant step towards professionalisation, the Academy of Oncology Nurse and Patient Navigators (AONN+) Foundation for Learning, Inc. achieved accreditation in the US to offer certification programs for oncology nurse navigators and oncology patient navigators in 2020.^12^ In 2022, the Professional Oncology Navigation Task Force in the US created professional practice standards to guide clinical and patient navigators, set benchmarks for employers, and inform policy- and decision-makers.^13^

Despite significant progress in defining and professionalising patient navigation in North America and beyond, such services do not always reach all patients who would benefit from them, and significant equity gaps persist in who receives navigation, with some tumour types and patient populations underserved.^3^ Additionally, there remains a critical gap in applying these professional frameworks and standards to other regions of the globe with different healthcare systems, cultures, and resources,^3^ as well as to volunteer or paid non-clinical (e.g., lay or peer) navigator roles. Consequently, international leaders have called for research to inform the global implementation of patient navigation,^3^^,^^14^ particularly in low- and middle-income countries and other settings where it is not yet a standard component of cancer care. To address this global gap, the Multinational Association of Supportive Care in Cancer (MASCC) and Flinders University created the Global Initiative to Advance Cancer Navigation for Better Outcomes (GINO).^15^ The aim of this study, the first GINO project, was to develop a global practice framework for patient navigation in cancer care, outlining the scope of patient navigation services across diverse healthcare systems. A practice framework has been defined as a ‘conceptual map’ that informs and supports practice by linking key foundational concepts with the practice interventions or activities that may be used to achieve desired outcomes.^16^ The purpose of our practice framework was to identify and organise the key interventions or activities provided as part of patient navigation in cancer care, to support ongoing global efforts to develop, implement and evaluate patient navigation programs and services. A practice framework for patient navigation in cancer care can be used by patient navigators or healthcare managers to implement or standardise navigation services across a health network, identify staffing models and training requirements, and support funding and resource allocation decisions.

## Methods

### Design

To ensure the framework was informed by evidence, stakeholder input, and contextual factors, we used a modified Delphi process^17^ to obtain expert consensus on the key practices or activities involved in providing patient navigation in cancer care. Delphi methods can involve experts anonymously completing iterative rounds of voting, which are interspersed with feedback based on the group's voting, until a pre-defined threshold for consensus is achieved.^18^ We followed the Recommendations for Conducting and Reporting of Delphi Studies (CREDES).^19^ Due to the international scope of the study, an asynchronous approach was undertaken.^20^

A GINO Steering Committee was formed to oversee the project design and interpretation of results, comprising 12 members from Australia, Canada, Mexico, Nigeria, and the US. The Steering Committee included the core research team (IR, FCW, CT, RC) and eight international advisors (DA, JB, AC, MC, MF, MJ, EM, ES, CT). Members were recruited through the Multinational Association of Supportive Care in Cancer (MASCC) and possessed clinical, research, policy, and advocacy expertise in patient navigation and have backgrounds in nursing, medical oncology, public health, pharmacy, behavioural science, consumer advocacy and lived experience. The Steering Committee conducted online bi-monthly meetings from January 2024 to progress the GINO project.

Additionally, a MASCC GINO Patient Navigation Working Group was established via an expression of interest to members of the MASCC Survivorship Study Group, to assist with recruiting international experts to the Delphi study.

The project involved two phases: (1) reviewing and summarising the literature on patient navigation in cancer care to generate a preliminary practice framework, and (2) reaching consensus on the included content and structure of the framework through a modified Delphi process.

### Phase 1: developing draft items for the practice framework

The research team reviewed literature on patient navigation in cancer care to identify key sources describing the scope of navigation, in terms of intervention components or activities that may be performed as part of navigation services, in the context of cancer care. Relevant literature was sought and used to develop initial items, however a systematic literature review was not conducted. A recently published umbrella review of systematic reviews of patient navigation^3^ was the basis of the evidence, which was complemented with a selection of other existing key frameworks including the Oncology Nursing Society core competencies for oncology nurse navigators,^8^ the oncology navigation professional practice standards,^13^ and the George Washington Cancer Institute's patient navigation framework.^9^^,^^11^ One member of the research team (IR) extracted a list of 124 patient navigation intervention components from these sources. Next, they refined this list to consolidate similar activities and organise them into higher-level practice items and domains. The Steering Committee provided feedback through an iterative process, until all members agreed on the structure and content of a preliminary list of 35 practice items across nine domains. This list informed the Delphi survey in Phase 2. The purpose of the Delphi rounds was then to identify any important items missing from this initial draft.

### Phase 2: achieving consensus on items for inclusion in the framework

Eligible participants were healthcare practitioners, researchers, policymakers, and patient partners, who were proficient in English and self-identified as having expertise in patient navigation. For the purpose of this study, we defined an expert as someone with either: ≥3 years clinical experience, ≥3 research outputs, or ≥3 years policy, leadership or advocacy experience relevant to patient navigation in cancer. As the Steering Committee had been involved in the development of the draft items, members were not invited to participate in the Delphi.

Potential participants were recruited using purposive and snowball sampling. A maximum-variation sampling approach^20^ was used to select a diverse set of participants from different disciplines, health system types, and world regions who had completed the expression of interest to join the MASCC GINO Patient Navigation Working Group. These participants were then invited to participate in the Delphi and forward the invitation to patient navigation experts from within their networks. The Steering Committee also identified a list of experts in patient navigation from the research literature, personal networks, and leadership of navigation organisations, initiatives, and programs (e.g., National Navigation Roundtable, AONN+, ONS) and approached them directly via email to participate. The invitation email briefly described the study aim, approach, and requirements for participation, and included a link to the participant information sheet, consent form, and online survey. While there are no concrete guidelines for the optimal number of Delphi survey participants to achieve consensus which can vary from 10 to 1000 in published studies, between 30 and 50 is considered optimal for a homogenous Delphi, although more may be required when looking at a broad heterogenous problem, such as navigation.^21^ Appropriate panel size with diverse representation of experts from different specialities and geographical distribution has been reported to improve generalisability.^21^ Therefore, we aimed to recruit at least 50 participants to ensure representation from people with diverse experience and across countries of different income categories.

The online survey was developed in Qualtrics and piloted with the Steering Committee prior to distribution. The survey included the list of practice items generated in Phase 1 organised by domain. At the commencement of the online survey, participants were provided a definition explaining that *navigation is generally understood as a set of individualised and patient-centred activities, distinguished from other cancer services by its focus on overcoming barriers to accessing timely and quality health care. The activities and tasks involved in navigation vary based on patient-, provider-, and system-level factors.* Participants were asked to rate each item using a five-point scale ranging from one (“Not important”) to five (“Critically important”). The survey included free-text items for participants to comment on or explain their ratings, suggest additional items for consideration, and provide feedback. Demographic items assessed participants’ age, gender, occupation, practice setting, country of practice, racial background,^22^ years of practice, and experience in patient navigation. The survey was developed in English and no translation in other languages was available.

The Delphi technique followed Hasson, Keeney and McKenna (2008) guidelines and involved asynchronous online survey rounds to enable participation from navigation experts from across the globe as the use of electronic surveys in a Delphi study can help with global representation of panel members.^17^^,^^21^ The Delphi was expected to be a minimum of two rounds, with further rounds possible if consensus was not reached during the final meeting.^17^ Participation involved completing two online surveys (Round 1 and Round 2), in which participants rated the importance of the practice items for inclusion in the framework. Potential participants were invited to Round 1 during July and August 2024. After collecting and analysing Round 1 responses, the Steering Committee revised and updated the Round 2 survey based on feedback and prepared a summary of results for participants. Round 2 was open to all participants who had completed Round 1. A member of the research team (IR) emailed participants with the summary of Round 1 results and revisions, along with instructions and a link to the Round 2 survey, which remained open for six weeks during November and December 2024. In the Round 2 survey, participants were asked to review the Round 1 results and re-rate all items. Providing participants with feedback on the group's voting and the opportunity to reconsider their initial judgements by re-rating items are considered important aspects of the Delphi method.^18^ Items that did not achieve consensus after Round 2 were reviewed and discussed in a consensus meeting.

Consensus was defined *a priori*. Items that received ratings of four or five out of five (i.e., “Very important” or “Critically important”) by ≥ 75% of participants and one or two (“Not important” or “Slightly Important”) by ≤ 15% of participants after Round 2 were deemed to have met consensus for inclusion. Although there are no definitive guidelines on how to define consensus in Delphi studies, these criteria are consistent with commonly used definitions of consensus in the literature.^23^

Ahead of the consensus meeting, Steering Committee members were provided with Round 2 ratings, qualitative feedback, and list of items that had not met consensus. Items were categorised based on whether they (1) reached consensus in Round 1, (2) received suggestions for rewording in Round 2, and (3) received a high proportion (≥20%) of scores of 1–2. Steering Committee members were first invited to provide written feedback via email and vote in an online survey on whether items should be discarded or considered further, based on the Delphi participants’ voting and qualitative feedback. This approach allowed Steering Committee members to share their views confidentially. Based on their responses, a list of items to be discarded, included with revisions, or included without revisions in the final framework was proposed. The consensus meeting was held online with members of the Steering Committee and facilitated by a member of the research team (IR), to discuss the proposed revisions and agree on the final included items.

#### Data analysis

Quantitative data were analysed using IBM SPSS Statistics for Windows, version 29.0 (IBM Corp., Armonk, NY, USA). Descriptive statistics were used to summarise results, including median scores and percentages of ratings between 1–2, and 4–5. Free-text responses were analysed using basic content analysis. Chi-square analyses were conducted to investigate potential differences in responses based on role (healthcare professionals, researchers, or consumers/advocates), resource setting (high or middle income), and navigation experience (clinical navigators, non-clinical navigators, or individuals without direct navigation experience).

### Ethics

The study received ethics approval from the Flinders University Human Research Ethics Committee (Project ID: 6799). Participants provided informed consent prior to completing the online survey.

### Role of the funding source

The funder of this study had no role in study design, data collection, data analysis, data interpretation, or writing of the report.

## Results

### Phase 1

A total of 35 unique practice items were identified from the key sources of literature.^3^^,^^8^^,^^9^^,^^11^^,^^13^ These items underwent coding and thematic analysis (IR) in order to classify them into nine distinct domains (identifying and addressing barriers to care; care planning, coordination and continuity, communication; support of direct clinical care provision; education; psychological, social, and emotional support; patient empowerment; advocacy; and health promotion) which were discussed and confirmed by the Steering Committee. While patient navigation in care is tailored to individual cancer types and care settings, the intervention components extracted from the source evidence were tumour agnostic with diverse applicability. For example, although items related to cancer screening were included, these would only be relevant for certain tumour types that have preventative screening programs.

### Phase 2

Following invitations sent to patient navigation experts across the globe, 81 respondents completed the Round 1 survey. These participants were then invited to Round 2, and 60/81 completed the survey (response rate = 74%). Table 1 presents the demographic characteristics of participants in Round 1 and Round 2 surveys. Participants in the Delphi survey rounds were mostly female (78%, n = 47/60), half were employed as healthcare practitioners (50%, n = 30/60), and the majority currently resided in a high-income country (65%, n = 39/60) (Table 1).

**Table 1:** Characteristics of Delphi participants in round 1 (n = 81) and round 2 (n = 60).

Variable	Characteristic	Round 1 n (%)	Round 2 n (%)
Gender			
	Woman	65 (80)	47 (78)
	Man	16 (20)	13 (22)
Age (years)			
	25–34	10 (12)	7 (12)
	35–44	22 (27)	18 (30)
	45–54	26 (32)	20 (33)
	55–64	14 (17)	10 (17)
	65+	9 (11)	5 (8)
Current role^a^			
	Healthcare practitioner	48 (59)	30 (50)
	Navigator (clinical)	9 (11)	9 (15)
	Navigator (non-clinical)	7 (9)	7 (12)
	Academic researcher	26 (32)	24 (40)
	Patient partner/patient advocate/consumer	11 (14)	6 (10)
	Advocate (non-patient)	10 (12)	4 (7)
	Other^b^	2 (2)	9 (15)
Clinical profession			
	Cancer patient navigator	3 (4)	0 (0)
	Clinical nurse specialist	5 (6)	3 (5)
	Medical oncologist	7 (9)	7 (12)
	Registered nurse	9 (11)	3 (5)
	Surgeon	3 (4)	2 (3)
	Other^c^	21 (26)	15 (25)
Work setting			
	Community health service	3 (4)	2 (3)
	Comprehensive cancer centre	17 (21)	11 (18)
	Public hospital or tertiary care centre	18 (22)	12 (20)
	Non-government organisation or civil society organisation	8 (10)	6 (10)
	University or academic/research institution	16 (20)	17 (28)
	Other^d^	11 (14)	6 (10)
Country			
	Australia	6 (7)	7 (12)
	Brazil	3 (4)	2 (3)
	Canada	12 (15)	10 (17)
	Croatia	5 (6)	3 (5)
	Egypt	3 (4)	3 (5)
	Mexico	4 (5)	4 (7)
	Nigeria	10 (12)	2 (3)
	Saudi Arabia	3 (4)	1 (2)
	United States of America	9 (11)	11 (18)
	Other^e^	24 (30)	18 (30)
World Bank country income group			
	Low	1 (1)	0 (0)
	Low middle	17 (21)	8 (13)
	Upper middle	18 (22)	13 (22)
	High	45 (56)	39 (65)
Racial background			
	Asian^f^	6 (7)	5 (6)
	Black or African American	11 (14)	5 (6)
	Hispanic or Latino	9 (11)	8 (13)
	Middle Eastern	4 (5)	1 (2)
	White or European	28 (35)	21 (35)
	Other^g^	23 (28)	19 (32)
Experience^h^			
	Worked in a clinical navigator role (e.g., nurse navigator)	23 (28)	13 (22)
	Worked in a non-clinical navigator role (e.g., (e.g., lay navigator)	12 (15)	7 (12)
	Educated/trained navigators	31 (38)	26 (43)
	Developed a navigation program, intervention, or service	44 (54)	32 (53)
	Managed a navigation program, intervention, or service	31 (38)	27 (45)
	Evaluated a navigation program, intervention or service	29 (36)	25 (42)
	Conducted research on navigation	33 (41)	28 (47)
	Developed guidance on navigation (e.g., core competencies)	27 (33)	17 (28)
	Had an advocacy role in navigation research, policy, or service provision	21 (26)	18 (30)
	Contributed as a patient partner/advocate to navigation programs, research, or policy	19 (23)	10 (17)
	Other^i^	4 (5)	3 (5)

*Note.* Categories and free-text responses selected by ≤ 2 participants in both rounds were combined into “Other”.

a Participants could select up to two options.

b Responses included health service leader, policy maker, research manager, navigation program coordinator, program lead.

c Responses included community health worker, gerontologist, haematologist, healthcare administrator, nurse practitioner, occupational therapist, paediatric oncologist, paediatrician, palliative care physician, pathologist, pharmacist, physiotherapist, psychologist, radiation oncologist, radiologist, social worker.

d Responses included other government department or agency, hospice/end-of-life care, health technology startup, pharmaceutical company, patient organisation.

e Responses included Argentina, Belgium, China, Colombia, Cyprus, Germany, Ghana, Indonesia, Ireland, Italy, Kenya, Malaysia, Philippines, Poland, Portugal, Rwanda, Thailand, Turkey, United Kingdom, Uruguay.

f Includes categories East Asian, South Asian, and Southeast Asian.

g Responses included Abuja, African, Alexandria, Amerasian, Balmoral, Cairo, Columbus, Dalhousie, Docklands, Dublin, East Berlin, Edmonton, Elizabeth Vale, Glenalta, Jewish, Melbourne, Mexico City, Mount Barker, Pasig City, Rome, São Paolo, Strombeek-Bever, Subang Jaya, Toronto, Washington, Westmount, Wlodowice, Wuxi.

h Participants could select multiple options.

i Responses included worked at a patient support helpline, founded the AONN+, funded navigation research and service provision, provided navigation as a caregiver, and published a paper on peer support.

After Round 1, 29 of the 35 items (83%) met consensus criteria for inclusion and six items (17%) did not. The Steering Committee revised 14 practice items (40%) based on qualitative feedback provided by participants. Revisions included using language to include the family and carer and providing additional clarification or examples of navigation activities (see Supplementary Table S1 for a summary of qualitative feedback and survey changes).

After Round 2, 27 of the 35 items (77%) met consensus criteria for inclusion and eight items (23%) did not reach consensus (Table 2). Two of the items that did not reach consensus received further suggestions for rewording to enhance clarity in Round 2. The first suggested revision aimed to clarify that a navigator's role in advocating for clinical trials focuses on informing and empowering individuals to support their participation, rather than engaging in general advocacy for clinical trial enrolment. The second revision proposed using more person-centred language instead of “treatment adherence” and clarified that the navigator's role is to identify and address barriers to achieving optimal treatment outcomes, while supporting treatment participation remains the responsibility of the clinical team.

**Table 2:** Summary of Delphi ratings in round 1 (n = 81) and round 2 (n = 60).

Domains and items	1–2	4–5	Consensus round 1	1–2	4–5	Consensus round 2
	(%)	(%)		(%)	(%)	
**Domain 1: identifying and addressing barriers to care**						
Identify patients' unmet needs and provide resources and referrals to address them	8	92	Criteria met	0	100	Criteria met
Assess factors impacting access to care for specific patient communities	4	92	Criteria met	0	98	Criteria met
Identify underserved and at-risk patients and locate resources to help them access care	4	93	Criteria met	0	92	Criteria met
Help patients, carers, and families navigate practical, financial, and administrative barriers to care (e.g., assisting with access to transportation, financial programs, or language services)	4	93	Criteria met	0	95	Criteria met
**Domain 2: care planning, coordination, and continuity**						
Support smooth transitions between care providers and settings, and across all phases of cancer care^a^	7	89	Criteria met	0	93	Criteria met
Support coordinated care with health care team (e.g., medical doctors, nurses, social workers, etc.)^a^	7	89	Criteria met	2	92	Criteria met
Promote timely access to and follow-up on diagnostic testing, treatment, and supportive care^a^	9	85	Criteria met	0	88	Criteria met
Support holistic care planning (i.e., in the context of functional status, employment, cultural considerations, health literacy, and psychosocial, reproductive, and spirituality needs)^a^	5	81	Criteria met	0	80	Criteria met
Maintain an up-to-date database of local, community, and national resources and establish relationships/linkages with the individuals/programs that provide these resources	6	82	Criteria met	3	78	Criteria met
**Domain 3: communication**						
Build trusting relationships with patients, carers, and families through effective listening and communication skills	6	88	Criteria met	0	98	Criteria met
Serve as a liaison between patients and providers and facilitate communication between team members and service providers	7	85	Criteria met	2	88	Criteria met
Facilitate patients', carers', and families' understanding and interpretation of information	6	93	Criteria met	0	100	Criteria met
**Domain 4: support of direct clinical care provision**						
Support patients' adherence to agreed-upon treatment plans^a^	10	72	No consensus	10	65	No consensus
Respond to patient questions and, where appropriate, refer patients to appropriate clinical colleagues	7	86	Criteria met	0	92	Criteria met
**Domain 5: education**						
Assess patients', carers', and families' health literacy and educational needs^a^	11	73	No consensus	8	61	No consensus
Provide tailored and culturally appropriate health education to patients, carers, and families^a^	6	83	Criteria met	0	92	Criteria met
Where appropriate, explain how the local cancer system works to patients, carers, and families, and educate them about the different roles of medical team members^a^	3	85	Criteria met	2	86	Criteria met
**Domain 6: health promotion**						
Assess patients' lifestyle and risk factors	6	69	No consensus	29	42	No consensus
Promote healthy lifestyle choices and support self-management^a^	4	76	Criteria met	7	80	Criteria met
Promote secondary cancer prevention and early detection behaviours^a^	14	70	No consensus	20	53	No consensus
Support and promote public health programs (e.g., cancer screening, genetic testing/counselling, vaccinations, mental health programs)^a^	14	74	No consensus	22	48	No consensus
**Domain 7: psychological, social, and emotional support**						
Screen patients, carers, and families for psychological distress^a^	6	85	Criteria met	2	86	Criteria met
Provide guidance to help patients, carers, and families cope with a cancer diagnosis and associated stress	4	85	Criteria met	3	92	Criteria met
Facilitate psychosocial assessment, referrals, and support to address patients', carers', and families' needs^a^	8	86	Criteria met	2	86	Criteria met
Facilitate access to psychosocial services to support transitions across all phases of cancer care	7	87	Criteria met	0	93	Criteria met
**Domain 8: patient empowerment**						
Support patients' self-advocacy skills	7	80	Criteria met	0	73	No consensus
Support and facilitate patients', carers', and families' understanding of care and participation in decision-making^a^	9	86	Criteria met	3	92	Criteria met
Assist patients in identifying and communicating their goals, preferences, concerns, and values to the healthcare team^a^	7	86	Criteria met	0	90	Criteria met
Provide individualised support for patients to achieve their health goals^a^	6	85	Criteria met	3	88	Criteria met
**Domain 9: advocacy**						Criteria met
Advocate for patients', carers', and families' care needs and preferences to be included in their treatment plan^a^	7	87	Criteria met	2	86	Criteria met
Advocate for the navigation profession	11	77	Criteria met	10	73	No consensus
Advocate for improved health services, programs, and resources^a^	7	88	Criteria met	5	83	Criteria met
Advocate for health policies and systems that protect and promote the interests of patients^a^	4	87	Criteria met	5	81	Criteria met
Advocate for health equity	4	86	Criteria met	2	90	Criteria met
Where applicable, advocate for patient participation in clinical trials	9	72	No consensus	20	59	No consensus

*Note: n*, number of complete responses.

a Item reworded for Round 2 based on participant feedback in Round 1. Consensus on inclusion was defined as ratings of 4–5 by ≥ 75% of participants and 1–2 by ≤ 15% of participants. Allocation of scores of 1–2 and 4–5 presented; ratings of 3 (“moderately important”) and 6 (“unsure/not my area of expertise”) not shown.

No significant differences were observed in the proportion of participants scoring items as very or critically important (scores of four or five), when comparing participants across professional roles (healthcare professionals, researchers, or consumers/advocates), resource settings (high or middle income), or navigation experience (clinical navigators, non-clinical navigators, or individuals without direct navigation experience); all *p* values > 0.05.

#### Consensus meeting

Eight items were reviewed in the consensus meeting. In the online survey, eight of nine Steering Committee members (89%) voted to discard three items from the health promotion domain that had not reached consensus in either round, had not received suggested revisions, and had received scores of 1–2 by ≥ 20% of participants. The majority of Steering Committee members (n = 5/9; 55%) voted to reconsider the remaining five items. The research team revised two items based on Round 2 feedback.

During the consensus meeting, the Steering Committee endorsed the inclusion of the two revised items, as well as the remaining three items. Key reasons included: (1) rewording improved the relevance of the revised items for navigation, (2) two items met the consensus threshold in Round 1 and were just 2% below the threshold of ≥75% rating four or five in Round 2, (3) qualitative feedback highlighted strong individual support for the inclusion of advocacy for the navigation profession and assessing health literacy/educational needs. An overarching theme of the discussion was that given the framework's aim was to define the scope of navigation practice, inclusivity was preferred over exclusion of practices still valued by the majority of participants.

**Fig. 1:**
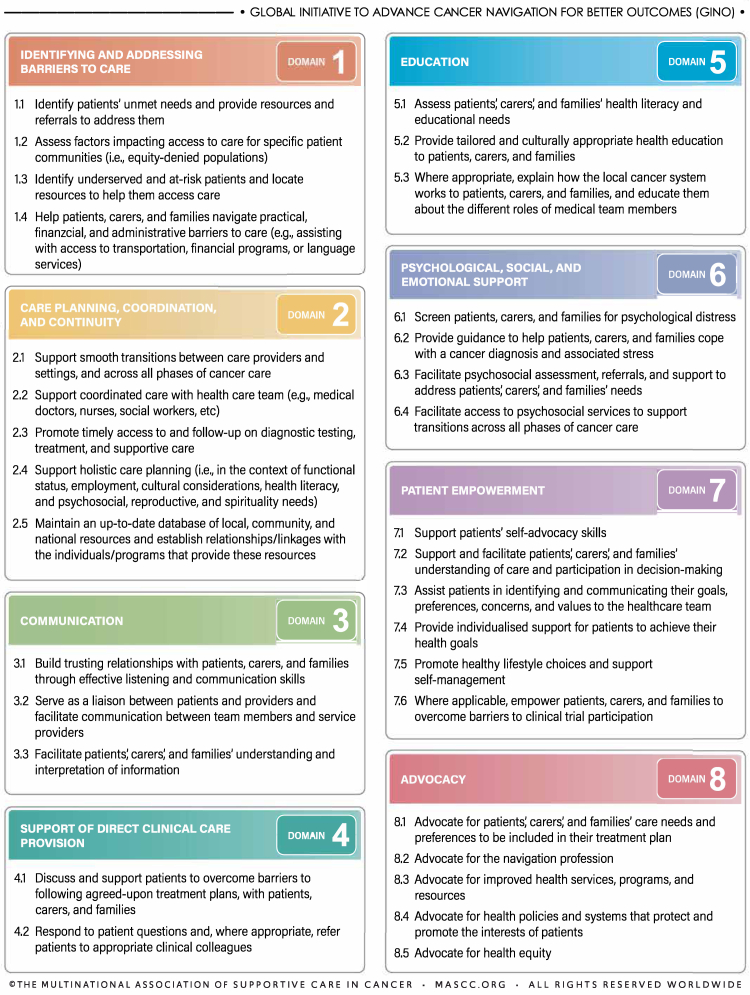
GINO practice framework for patient navigation in cancer care.

Following expert review, 32 practice items were included in the final GINO practice framework (Fig. 1). Due to the removal of three out of four items from the health promotion domain, this domain was also removed and the remaining items reclassified into the patient empowerment domain. Supplementary Table S2 presents the included domains and items in the GINO framework, mapped to the original sources of guidance on navigation to provide an indication of their coverage.

## Discussion

By developing a consensus-based, global practice framework for patient navigation in cancer care, this study obtained important insights into how patient navigation is conceptualised by experts across diverse health systems, resource settings, and global environments. A key finding was the strong consensus on the importance of identifying and addressing barriers to care, aligning with foundational definitions of patient navigation,^1^^,^^24^ and core objectives of navigation services aiming to support equitable care across the cancer continuum.^3^ Items within this domain (e.g., helping patients, carers and families navigate practical and administrative barriers to care) received strong endorsement, reflecting their perceived centrality to patient navigation. Supporting not only patients, but also their families and carers, was a clear theme in both ratings and qualitative feedback. Several items were revised between rounds to reflect inclusive language and the role of navigation services in supporting carers and families, highlighting the broader impact of cancer on patients’ support networks,^25^ and importance of navigation services that address their needs.

Other practices that received high endorsement (90–100%) were supporting smooth transitions and coordinated care; building trusting relationships; facilitating understanding of information; responding to questions and referring to appropriate colleagues; providing tailored health information; helping individuals cope with a cancer diagnosis; facilitating access to psychosocial services; supporting participation in decision-making; and advocating for health equity. This strong endorsement of practices centred on communication, coordinated care, and helping individuals feel informed, empowered and supported throughout their cancer care highlights the multidimensional nature of patient navigation as a relational, person-centred intervention.^2^^,^^26^ These findings reinforce that patient navigation not only addresses barriers to care but entails critical functions for enhancing experiences of care across the cancer continuum.^2^^,^^9^

The GINO practice framework for patient navigation in cancer care was developed to complement existing guidance, primarily originating from North America and focused on specific navigator roles. This framework differs from previously available frameworks in that, it pragmatically focuses on practical activities, rather than practice standards of “navigators”.^13^ This framework should not be used as a one-size-fits-all approach as its comprehensive nature means that not all activities are relevant to all programs across all context and regions. The practices within the GINO framework were drawn from various sources, resulting in considerable overlap. However, no single source encompasses all the domains and practices ultimately included in the GINO framework (see Supplementary Table S2). Likewise, the GINO framework does not incorporate many detailed or role-specific tasks outlined in these sources. These distinctions reflect the intended scope of the GINO framework, which seeks to identify and organise the key activities involved in delivering navigation services. The knowledge, skills, and abilities described in core competency frameworks were beyond the scope of this work. In contrast to the patient navigation framework developed by Willis et al. (2013), which specifies responsibilities by navigator type to support competency development,^9^ the GINO framework was created for more flexible adoption. It acknowledges that patient navigation interventions may be provided by individuals with varied professional backgrounds and training, influenced by the local context, health system, and resources. Importantly, the framework is not meant to define a minimum set of activities required for a program to align with best practice. Instead, it offers a descriptive overview of patient navigation components to inform service design across different settings. The broad nature of the domains that are relevant across many chronic diseases (identifying and addressing barriers to care; care planning, coordination and continuity, communication; support of direct clinical care provision; education; psychological, social, and emotional support; patient empowerment and advocacy) means that these standards could be easily adapted for patient navigation services beyond cancer care.

The framework has wide-ranging potential applications and implications for research, practice, and policy. For researchers, it provides a structured foundation to guide further inquiry into the mechanisms and outcomes of navigation interventions. For navigators and navigation program developers, it offers a concise overview of key activities or practices involved in navigation, which can support service design, implementation, and evaluation, and contribute to standardisation of competencies. It may also help to harmonise navigation practices across different countries and health systems while allowing for contextual adaptation. For policymakers, the framework provides further support for efforts to formalise and fund navigation services as a recognised component of cancer care, and to ensure appropriate workforce development. GINO is a three-stage project, the second stage is to develop a set of quality indicators to support service delivery, and the third stage is the development of a global community of practice.^15^ This group aims to unify, evidence-based guiding principles that support local implementers of patient navigation worldwide.^27^

This study highlights several important directions for future research. First, there remains limited clarity around the scope of patient navigation roles in some countries. Further research is needed to support role optimisation and effective interprofessional collaboration, for example, by exploring how lay or peer navigators can work alongside clinical navigators to maximise efficiency and minimise role overlap. Additional exploration is also warranted to better understand the role of health promotion within navigation and how it intersects with other roles, such as health coaching. To develop meaningful guidance for implementation in diverse settings, it will be essential to engage with stakeholders across different countries and communities to understand their specific needs for navigation services and how these roles can be adapted to fit within local health systems. Moreover, research should evaluate the effectiveness of different navigation components to determine which activities have the greatest impact on outcomes, in which contexts, and at which points along the cancer care continuum. The second part of this project, to develop a globally recognised comprehensive and inclusive list of quality indicators for patient navigation, will help to address some of these gaps. These indicators will be able to be used by administrators and navigators to help to both develop and evaluate navigation services. Finally, there is also a need for robust evaluation of training models and implementation strategies to understand how navigation practices can be effectively scaled and sustained across diverse healthcare and cultural settings.

Several limitations should also be acknowledged. As a survey-based study, the findings of this work relied on self-reported data and may be subject to response bias. The use of purposive sampling, while appropriate for Delphi studies, limits generalisability of results. Although we recruited a diverse sample, with varied navigation experience and geographic representation, it may not be representative of the broader international community engaged in navigation. Particularly, there is minimal representation from primary care. Finally, while the Delphi technique is a widely accepted method for achieving expert consensus, the absence of formal methodological standards introduces potential for variability in its application, which may affect the replicability of findings.

Despite limitations, this study was the first effort to engage global experts in developing a practice framework for patient navigation in cancer care. The resulting GINO framework reflects the collective expertise of international stakeholders and offers an adaptable tool to help guide the design, implementation, and evaluation of patient navigation services. By focussing on the interventions and activities that characterise patient navigation, rather than specific competencies or roles, the framework complements existing guidance and accommodates variation in how patient navigation is delivered around the world. It provides a foundation for strengthening practice globally and sets the stage for future research to refine roles, assess impact, and guide the integration of patient navigation into existing models of care.

## Contributors

IR, FCW, CT, DA, JLB, AC, MC, MIF, JM, EM, ES, CT, and RJC were responsible for the conceptualisation, methodology, investigation, and project administration. IR was responsible for data collection, and CT, FCW verified the underlying data. IR performed formal analysis and wrote the original draft. RJC supervised the project. IR, FCW, CT, DA, JLB, AC, MC, MIF, JM, EM, ES, CT, and RJC critically revised the manuscript. IR, FCW, CT, and RJC had full access to the study data. All authors read and approved the final version of the manuscript.

## Data sharing statement

Data from this study are available from the corresponding author on reasonable request.

## Declaration of interests

Novartis provided funding for this study. All aspects of study design, conduct, data analysis, interpretation, and publication were undertaken independently by the authors. Dawn Aubel was employed by Novartis when the research commenced; however, Novartis had no involvement in, or influence over, the study design, data collection, analysis, interpretation, or the decision to publish the findings and Novartis received no financial gain from providing this funding.

## Appendix A Supplementary data

Supplementary Material

Appendix Group Author List
