# Supplementary material for: Developing a practice framework for patient navigation in cancer care: a Global Initiative to Advance Cancer Navigation for Better Outcomes (GINO) project
Source: eClinicalMedicine. 2026 Feb 23;93:103808. doi: 10.1016/j.eclinm.2026.103808 (PMC12973518; doi:10.1016/j.eclinm.2026.103808)
Supplement: Supplementary Material [file mmc1.docx]

**Supplementary material**

**Table of contents**

| Supplementary Table 1: Summary of qualitative feedback and changes to items after Round 1 | **2** |
| --- | --- |
| Supplementary Table 2: Items included in the GINO practice framework for patient navigation, mapped to existing sources on patient navigation | **6** |

**Supplementary Table 1.** Summary of qualitative feedback and changes to items after Round 1

| **Domain** | **Feedback summary** | **Changes** |
| --- | --- | --- |
| **Domain 1: Advocacy** | - Advocating for system change, the profession, and participation in clinical trials are potentially beyond the scope of navigation. - Involving patients from LMICs in clinical trials is very important. - Advocacy tasks are overarching and can be separate from patient-facing navigation work. It is important this work is done, but not necessarily by people or organisations providing navigation services. - Important to mention the patient and their family member or caregiver throughout the framework. | - **Domain 1** (Advocacy) moved to the end of the framework (Domain 9) to help separate advocacy tasks from patient-facing tasks. - **Bullets 1 and 4**: Added “carers and families” (as well as in relevant items in Domain 2, 5, and 7) |
| **Domain 2: Identifying and addressing barriers to care** | - A major focus of navigation, especially in LMICs. - Navigators should focus on the individual, whilst keeping note of factors impacting specific communities and raising these with individuals who have influence in the healthcare and policy domain. - Does not need to be done by an individual, although it can be, but could also be a digital tool to support navigation. - These issues may be dealt with by social workers and patient relations officers. | - N/A |
| **Domain 3: Care planning, coordination, and continuity** | - Role of the navigator in some categories is more on the “support” side than the “facilitate” side. - Crucial in healthcare systems where care is not comprehensive, and patients have to go to different places to access services. - The extent of integration with the clinical team will determine how much they can contribute to 2 and 3. The navigator may not be able to promote timely follow-up on diagnostic, testing, treatment and supportive care services, but could potentially facilitate it by encouraging the patient to find and access follow-up care services. - Mainly the role of HCPs and institutions, although this is often lacking, and navigation services can fill a gap. - The database needs to be part of the health data ecosystem. - Mention timely performance of or access to diagnostic/testing services if required, and subject to availability. | - **Bullets 1, 2, and 4:** Deleted “and facilitate”, e.g.,   *Support ~~and facilitate~~ smooth transitions between care providers and settings, and across all phases of cancer care.*   - **Bullet 3:** Added “access”   *Promote timely access to and follow-up on diagnostic testing, treatment, and supportive care.* |
| **Domain 4: Communication** | - Communication is key and the navigator has a critical role in upholding patient ownership of their care, particularly in certain cultures. - Liaising between patients and providers would be very time consuming, so 1 and 3 are the basis to start from. - Liaison should not be necessary if HCPs collaborate. It is more important to give patients/carers tools for better communication on their end. - All are critical and dependent on roles, so the framework should function in a matrix team philosophy. - Interpretation of information is with clinicians. | - Addressed scope and function of the framework in the introduction |
| **Domain 5: Support of direct clinical care provision** | - As most navigation programs are nurse run and don't have physician oversight, onus should not be on navigation programs but the most responsible practitioner. - Need to ensure patients are aware of team members and who to call re treatment or symptom management concerns. - While important, patients and providers must be responsible for adherence to treatment plans as well. - Not sure how supporting treatment adherence could be achieved in practice, as it might require regular contact and commitment to a patient. It would be good if it is possible. - A complex issue involving patient, care givers, pharmacist, nurses and how flexible and well-established is the referring system. - Patients bond with their navigator and confide in them things they don't tell their treatment team. Lack of adherence is an example of that. - Digital solutions can also support this. | - **Bullet 1:** Reworded so that the focus is more clearly on supporting patients to adhere to treatment plans agreed with their clinical team:   *~~Monitor and~~ support patient’s adherence to agreed-upon treatment plans ~~(e.g. symptom management)~~.* |
| **Domain 6: Education** | - A system-tailored navigation program is crucial to factor in the local environment. - In Nigeria, clinical education should be done by the HCPs. - Education needs to be delivered once the navigator understands the level of patients' medical literacy and desire for knowledge, some patients do not want to know. Cultural references can be missed if the navigator is not well trained. - This in itself is a full-time job and complex issue with variable practices. - Orienting to the health system can be overwhelming. Its best to give an overview then as each phase of treatment is about to begin give details about the treatment team managing that specific phase of care. - There should be some thought about risk management from a clinical, social and psychosocial perspective too, Navigators should be managing caseloads based on consumers being risk-stratified, so people get the support when they need it rather than all patients getting the same. | - **Bullet 1:** Added “carers and families” and “health literacy”   *Assess patients’, carers’, and families’ health literacy and educational needs.*   - **Bullet 2 2:** Added “tailored”   *Provide tailored and culturally appropriate health education to patients, carers, and families.*   - **Bullet 3:** Added “where appropriate”   *Where appropriate, explain how the local cancer system works to patients, carers, and families, and educate them about the different roles of medical team members.* |
| **Domain 7: Health promotion** | - Cancer recurrence should be included. - Include vaccination campaigns, mental health programs, and prevention strategies. - Depends largely on type of navigation – a lay vs clinical navigator would approach this differently. - All these activities are very important but may fall under a health coach role as opposed to a navigator role. Unless we are proposing that the navigator role includes health coaching. - If navigation refers to the practice of providing personalized support to people with cancer, to help them access healthcare then the above are not as relevant in their work with patients. - Patients using navigation services already have cancer, so the service should be focused on that. Prevention is a whole different domain that requires different expertise. - As a clinician you may give advice but what are boundaries between a family doctor and a cancer specialist. - Commonly this is done by community outreach navigators. Once diagnosed, the navigator involved with that patient's care and treatment does an assessment for that specific patient and their family. - The framework needs to include all of this but there is a partnership philosophy that needs to be adopted with organisations that do this already. - This area is with clinicians, and nurse specialists. - Navigators can also provide support for self-management and patient empowerment. | - Bullet 1: Added “support”   *Promote healthy lifestyle choices and support self-management*   - **Bullet 3:** Specified “secondary”   *Promote secondary cancer prevention and early detection behaviours*   - **Bullet 4:** Added suggested examples   *Support and promote public health programs (e.g., cancer screening, and genetic testing/counselling, vaccinations, mental health programs)*   - Addressed issue of role overlap in introduction |
| **Domain 8: Psychological, social, and emotional support** | - A focus on psychosocial oncology is crucial for navigation but the resources must be available to respond to screening - which is not the case now. - Crucial to have a strong psychosocial colleague on navigation team. - Screening for distress should fall under the primary objective of identifying needs. - This domain is very important and should be done at the start of the relationship with the patient for a holistic approach to care. - Support active presence of a psychologist and social worker in each care set up. - Include the family caregiver. - While screening is done by navigator, the rest is by nurse specialists and clinicians. | - Bullets 1 and 3: Added “Carers and families” |
| **Domain 8: Patient empowerment** | - If goals include health behaviour goals, may want to reflect on how the navigator role overlaps/complements the health coach role. - In some communities asking for patient preferences can be seen as weakness or lack of expertise from the HCP. - Important for all patients but especially those with stage 4 disease. Doctors will offer/provide another treatment no matter how futile. We must advocate for the patient. At a minimum, the navigator should remain in touch with the patient and their family. - The individualized support sounds lovely, but possibly resource intensive. - Mutual relationships needed to ensure expectations of the health service are clear. Patients should be educated on the journey they will take through their cancer service, when they can expect results, decision choices and interactions so they can self-advocate and be empowered to ask the right questions. This framework should also include coaching to improve patient wellbeing and empowerment to so they can own their story and manage their own journey. - Individualized goals of care are done by clinicians. | - **Bullet 1:** Deleted “foster”   *~~Foster and~~ support patients’ self-advocacy skills*   - Addressed issue of role overlap in introduction |

**Supplementary Table 2.** Items included in the GINO practice framework for patient navigation, mapped to existing sources on patient navigation

|  | ONSPP | ONNC | GWCI CCOPN | PNF |
| --- | --- | --- | --- | --- |
| ***DOMAIN 1: IDENTIFYING AND ADDRESSING BARRIERS TO CARE*** |  |  |  | X |
| *Identify patients’ unmet needs and provide resources and referrals to address them* | X | X |  | X |
| *Assess factors impacting access to care for specific patient communities* | X |  | X | X |
| *Identify underserved and at-risk patients and locate resources to help them access care* | X |  | X | X |
| *Help patients, carers, and families navigate practical and administrative barriers to care (e.g., assisting with access to transportation, financial programs, or language services)* | X | X | X | X |
| ***DOMAIN 2: CARE PLANNING, COORDINATION, AND CONTINUITY*** | X | X |  | X |
| *Support smooth transitions between care providers and settings, and across all phases of cancer care* | X | X | X |  |
| *Support coordinated care with health care team (e.g., medical doctors, nurses, social workers, etc.)* | X | X |  | X |
| *Promote timely access to and follow-up on diagnostic testing, treatment, and supportive care* | X | X |  |  |
| *Support holistic care planning (i.e., in the context of functional status, employment, cultural considerations, health literacy, and psychosocial, reproductive, and spirituality needs)* | X | X |  |  |
| *Maintain an up-to-date database of local, community, and national resources and establish relationships/linkages with the individuals/programs that provide these resources* | X | X |  |  |
| ***DOMAIN 3: COMMUNICATION*** | X | X | X | X |
| *Build trusting relationships with patients, carers, and families through effective listening and communication skills* | X | X | X |  |
| *Serve as a liaison between patients and providers and facilitate communication between team members and service providers* |  | X | X |  |
| *Facilitate patients’, carers’, and families’ understanding and interpretation of information* |  |  |  | X |
| ***DOMAIN 4: SUPPORT OF DIRECT CLINICAL CARE PROVISION*** |  |  |  |  |
| *Discuss and support patients to overcome barriers to following agreed-upon treatment plans, with patients, carers, and families* | X |  | X |  |
| *Respond to patient questions and, where appropriate, refer patients to appropriate clinical colleagues* | X |  |  |  |
| ***DOMAIN 5: EDUCATION*** | X | X |  | X |
| *Assess patients’, carers’, and families’ health literacy and educational needs* |  | X | X | X |
| *Provide tailored and culturally appropriate health education to patients, carers, and families* | X | X |  | X |
| *Where appropriate, explain how the local cancer system works to patients, carers, and families, and educate them about the different roles of medical team members* |  | X | X |  |
| ***DOMAIN 6: PSYCHOLOGICAL, SOCIAL, AND EMOTIONAL SUPPORT*** | X |  |  | X |
| *Screen patients, carers, and families for psychological distress** | X |  |  | X |
| *Provide guidance to help patients, carers, and families cope with a cancer diagnosis and associated stress* | X | X |  | X |
| *Facilitate psychosocial assessment, referrals, and support to address patients’, carers’, and families’ needs* | X | X |  | X |
| *Facilitate access to psychosocial services to support transitions across all phases of cancer care* | X | X |  | X |
| ***DOMAIN 7: PATIENT EMPOWERMENT*** |  |  |  | X |
| *Support patients’ self-advocacy skills* | X | X | X |  |
| *Support and facilitate patients’, carers’, and families’ understanding of care and participation in decision-making* | X | X |  | X |
| *Assist patients in identifying and communicating their goals, preferences, concerns, and values to the healthcare team* | X | X | X | X |
| *Provide individualised support for patients to achieve their health goals* | X |  |  | X |
| *Promote healthy lifestyle choices and support self-management* | X |  | X |  |
| *Where applicable, empower patients, carers and families to overcome barriers to clinical trial participation* |  |  |  |  |
| ***DOMAIN 8: ADVOCACY*** | X |  |  | X |
| *Advocate for patients’, carers’, and families’ care needs and preferences to be included in their treatment plan* |  |  |  | X |
| *Advocate for the navigation profession* | X |  |  |  |
| *Advocate for improved health services, programs, and resources* | X |  | X |  |
| *Advocate for health policies and systems that protect and promote the interests of patients* | X |  |  |  |
| *Advocate for health equity* | X |  |  |  |

Note. ONSPP = Oncology Navigation Standards of Professional Practice, ONNC = Oncology Nurse Navigator Competencies, GW = George Washington Cancer Institute Core Competencies for Oncology Patient Navigators, PNF = Patient Navigation Framework.
