# Supplementary material for: Developing a practice framework for patient navigation in cancer care: a Global Initiative to Advance Cancer Navigation for Better Outcomes (GINO) project
Source: eClinicalMedicine. 2026 Feb 23;93:103808. doi: 10.1016/j.eclinm.2026.103808 (PMC12973518; doi:10.1016/j.eclinm.2026.103808)
Supplement: Appendix Group Author List [file mmc2.docx]

Appendix – Group author list

**MASCC GINO Patient Navigation Working Group:** Oluwaseyifunmi Andi Agbejule,^1^ Muna Alkhaifi,^2^ Cristiane Bergerot,^3^ Darcy Burbage,^4^ Andreai Capela,^5^ Yin Ting Cheung,^6,7^ Niharika Dixit,^8,9^ Carolyn Ee,^1^ Kristen Haase,^10,11^ Nicolas H. Hart,^1,12^ Darren Haywood,^12,13^ Ria Joseph,^1^ Debbie Kirk,^14,15^ Stefano Magno,^16,17^ Aalaa Mahmoud,^18^ Duska Petranovic,^19,20^ Dagmara Poprawski,^21,22^ Emad Shash,^23^ Sandra Sonego^24^

**Affiliations:**

1. Caring Futures Institute, Flinders University, Adelaide, South Australia, Australia
2. Department of Medical Oncology & Hematology, Odette Cancer Centre, Sunnybrook Health Science Centre, University of Toronto, Toronto, Ontario, Canada
3. Oncoclinicas&Co—Medica Scientia Innovation Research (MEDSIR), Sao Paulo, SP, Brazil
4. Oncology Clinical Nurse Specialist, Newark, Delaware, United States
5. Oncology Department, Unidade Local de Saúde de Gaia e Espinho, Vila Nova de Gaia, Portugal
6. Faculty of Medicine, School of Pharmacy, The Chinese University of Hong Kong, Hong Kong, China
7. Hong Kong Hub of Paediatric Excellence, The Chinese University of Hong Kong, Hong Kong, China
8. University of California San Francisco, San Francisco, California, United States
9. Zuckerberg San Francisco General Hospital, San Francisco, California, United States
10. UBC School of Nursing, The University of British Colombia, Vancouver, British Colombia, Canada
11. BC Cancer Research Institute, Cancer Control, Vancouver, British Colombia, Canada
12. Human Performance Research Centre, INSIGHT Research Institute, Faculty of Health, University of Technology Sydney (UTS), Sydney, New South Wales, Australia
13. St. Vincent’s Hospital Melbourne, Fitzroy, Victoria, Australia
14. School of Nursing and Midwifery, Edith Cowan University, Bunbury, Western Australia, Australia
15. School of Nursing and Midwifery, La Trobe University, Bundoora, Victoria, Australia
16. Medical Oncology, IRCCS Sacro Cuore Don Calabria Hospital, Negrar di Valpolicella, Verona, Italy
17. Medical Oncology Unit, IRCCS Ospedale Policlinico San Martino, Genova, Italy
18. Department of Clinical Oncology, Aswan Cancer Center, Aswan, Egypt
19. Department of Hematology, Clinical Hospital Center Rijeka, Krešimirova 42, 51000 Rijeka, Croatia
20. Faculty of Medicine Rijeka, University of Rijeka, Braće Branchetta 20, 51000 Rijeka, Croatia
21. Department of Oncology, King Faisal Specialist Hospital & Research Centre, Riyadh, Kingdom of Saudi Arabia
22. College of Medicine and Public Health, Flinders University, Adelaide, South Australia, Australia
23. Medical Oncology Department, National Cancer Institute, Cairo University, Giza, Egypt
24. Translational Health Research Institute, Western Sydney University, Sydney, New South Wales, Australia

| **First names** | **Surnames** |
| --- | --- |
| Oluwaseyifunmi | Agbejule |
| Muna | Alkhaifi |
| Cristiane | Bergerot |
| Darcy | Burbage |
| Andreai | Capela |
| Yin Ting | Cheung |
| Niharika | Dixit |
| Carolyn | Ee |
| Kristen | Haase |
| Nicolas | Hart |
| Darren | Haywood |
| Ria | Joseph |
| Debbie | Kirk |
| Stefano | Magno |
| Aalaa | Mahmoud |
| Duska | Petranovic |
| Dagmara | Poprawski |
| Emad | Shash |
| Sandra | Sonego |
